# CircAGFG1 drives metastasis and stemness in colorectal cancer by modulating YY1/CTNNB1

**DOI:** 10.1038/s41419-020-2707-6

**Published:** 2020-07-17

**Authors:** Lei Zhang, Xiaoqiao Dong, Bo Yan, Wenhua Yu, Letian Shan

**Affiliations:** 1 School of Biological and Chemical Engineering, Zhejiang University of Science and Technology, Hangzhou, 310023 Zhejiang China; 2 Department of Neurosurgery, Affiliated Hangzhou First People’s Hospital, Zhejiang University School of Medicine, Hangzhou, 310006 Zhejiang China; 3 the First Affiliated Hospital, Zhejiang Chinese Medical University, Hangzhou, 310053 Zhejiang China

**Keywords:** Cancer prevention, Colorectal cancer

## Abstract

Colorectal cancer (CRC) is a common malignancy with high occurrence and mortality worldwide. In recent years, the overall survival rate of CRC patients has been improved because of the advances in early diagnosis and therapy. However, the prognosis of CRC patients at the advanced stage is still poor due to high recurrence rate and metastasis. The function of circular RNA (circRNA) ArfGAP with FG repeats 1 (circAGFG1) has been explored in non-small-cell lung cancer and triple-negative breast cancer. Nevertheless, its role in CRC is not clear. In this study, circAGFG1 was upregulated in CRC cell lines. CircAGFG1 silencing significantly suppressed cell proliferation, migration, invasion, and stemness, while promoted cell apoptosis in CRC. Meanwhile, we found that circAGFG1 also accelerated CRC tumor growth and metastasis in vivo. Importantly, circAGFG1 activated Wnt/β-catenin pathway through regulating CTNNB1. Afterwards, YY1 was found to transcriptionally activate CTNNB1. Furthermore, circAGFG1 directly sponged miR-4262 and miR-185-5p to upregulate YY1 expression. Eventually, rescue assays demonstrated that the effect of circAGFG1 silencing on CRC cell functions was observably reversed by upregulating YY1 or CTNNB1. In brief, our findings uncovered that circAGFG1 modulated YY1/CTNNB1 axis to drive metastasis and stemness in CRC by sponging miR-4262 and miR-185-5p.

## Introduction

Colorectal cancer (CRC) is a common malignancy with a high risk of occurrence and death worldwide^1–3^. With the development of medical technology, the methods of early diagnosis and therapy for CRC have been improved. Accordingly, the overall survival rate of CRC patients has been improved. However, owing to the high recurrence rate and metastasis of CRC, the prognosis of patients at the advanced stage is still unsatisfactory^4,5^. The molecular mechanisms underlying CRC development are complex, and many different factors affect the biological processes of CRC. Thus, it is very necessary to reveal the underlying molecular mechanism and discover more effective biomarkers for improving the prognosis of CRC patients.

Circular RNAs (circRNAs) are characterized by the covalently closed circular structures, which are regarded as non-protein coding transcripts. CircRNAs have been discovered as critical regulators in cancer progression^6,7^. Increasing studies have demonstrated that circRNAs can regulate the biological behaviors of diverse cancers. For example, hsa_circ_0078602 acts as a prognostic biomarker for hepatocellular carcinoma patients^8^. CircNF1 regulates miR-16 expression to promote gastric cancer progression^9^. Hsa_circ_0052112 modulates miR-125a-5p expression, thus accelerating cell migration and invasion in breast cancer^10^. CircRNAs also plays pivotal regulatory roles in CRC. As reported, circVAPA is upregulated and exhibited oncogenic property in CRC by sponging miR-101^11^. Hsa_circ_0136666 facilitates cell proliferation and invasion through targeting SH2B1 in CRC^12^. CircITGA7 modulates the Ras pathway and upregulated ITGA7 to suppress CRC growth and metastasis^13^. CircHIPK3 acts as miR-7 sponge to drive CRC development^14^. CircRNA derived from ArfGAP with FG repeats 1 (circAGFG1) has been reported to accelerate epithelial-mesenchymal transition (EMT) of non-small-cell lung cancer by sponging miR-203 and upregulating ZNF281 expression^15^. Moreover, circAGFG1 functions as a sponge for miR-195-5p to facilitate triple-negative breast cancer progression^16^. However, the function and mechanism of circAGFG1 in CRC remain unclear.

Previous studies have shown that Wnt/β-catenin signaling pathway participates in the regulation of tumor growth, stemness, tumor microenvironment and metabolism, and plays critical regulatory roles in various malignant tumors^17,18^. Existing evidence has confirmed that Wnt/β-catenin signaling pathway is involved in the tumorigenesis of CRC. For example, EGFL6 enhanced CRC cell proliferation via activating Wnt/β-catenin pathway^19^. Targeting Wnt/β-catenin signaling pathway has been certified to have potential value for the treatment of CRC^20^. HES6 is a biomarker of poor prognosis, whose upregulation aggravates metastasis in CRC through the Wnt/β-catenin signaling pathway^21^. CTNNB1 gene (Gene ID: 1499), also known as β-catenin, is a pivotal effector in canonical Wnt signaling pathway. The role of CTNNB1 in tumors was also widely reported. For instance, knockdown of long non-coding RNA (lncRNA) SNHG5 ameliorates glioma malignant cellular phenotypes via inhibiting Wnt/CTNNB1 pathway^22^. LncRNA DANCR enhances stemness features via upregulation of CTNNB1 in hepatocellular carcinoma^23^. However, the potential molecular mechanism by which circAGFG1 regulates CTNNB1 has not been explored in cancers, including CRC.

In this work, we probed the function and mechanism of circAGFG1 in CRC.

## Materials and methods

### Cell lines and treatment

Human colon epithelial cell line (NCM460) and CRC cell lines (SW480, HCT116, SW620 and HT-29) were obtained from Shanghai Institute of Biochemistry and Cell Biology (Chinese Academy of Sciences, Shanghai, China). Cells were allowed to grow in a 37 °C incubator with 5% CO_2_. Dulbecco’s Modified Eagle Medium (DMEM; Gibco, Grand Island, NY, USA) with 10% fetal bovine serum (FBS; Gibco) and 1% antibiotics (Gibco) were utilized for cell culture. Wnt/β-catenin pathway activator LiCl, AKT activator SC79 and Notch pathway activator Jagged1 were obtained from Sigma-Aldrich (St. Louis, MO, USA). Cells were incubated with the addition of these agents for signaling pathway activation assay.

### Quantitative real-time polymerase chain reaction (qRT-PCR)

Total RNAs were extracted from cells with TRIzol reagent (Invitrogen, Carlsbad, CA, USA) and reversely transcribed into complementary DNAs (cDNAs). SYBR Green PCR Master Mix (Invitrogen) was then used for qRT-PCR analysis on Step-One Plus System (Applied Biosystems, Foster City, CA, USA). RNA relative expression was calculated by 2^−ΔΔCT^ method, with GAPDH or U6 as the control.

### Nucleic acid electrophoresis

First, cDNA or genomic DNA (gDNA) extracted from cells underwent PCR analysis using specific convergent or divergent primers designed for circAGFG1 or GAPDH as the control. Agarose gel and Tris-EDTA (TE) buffer (Thermo Scientific, Waltham, MA, USA) were applied for the detection of PCR products, with DL600 (KeyGen, Nanjing, China) as the DNA marker. PCR products were separated by electrophoresis, followed by ultraviolet irradiation.

### Actinomycin D (ActD) and ribonuclease (RNase R) treatment

SW480 and HCT116 cells were treated with 2 mg/ml ActD (Sigma-Aldrich) to inhibit RNA synthesis. After 0, 4, 8 or 12 h or incubation, qRT-PCR was conducted to detect the levels of remaining circAGFG1 and AGFG1 messenger RNA (mRNA). For RNase R treatment, RNAs extracted from cells were treated with 3 U/μg of RNase R and then underwent qRT-PCR. The levels of circAGFG1 and AGFG1 mRNA were evaluated.

### Human tissue samples

Ethical approval for the study was acquired from the Ethics Committee of Affiliated Hangzhou First People’s Hospital, Zhejiang University School of Medicine and written informed consents were obtained by all participants. Tumor specimens and adjacent normal tissues (*n* = 30) were collected from recruited CRC patients, including 11 female patients and 19 male patients. Age range of patients enrolled in this study were between 36 and 71. Thirty patients with lymph node metastasis, while 17 of them without lymph node metastasis. Twelve patients were in I–II stage (TNM stage) and 18 of them were in III–IV stage. All these patients did not receive any preoperative treatment from 2013 to 2018, and preserved in liquid nitrogen at −80 °C.

### Cell transfection

The short hairpin RNAs (shRNAs), including sh-circAGFG#1/2, sh-YY1#1/2, sh-ER-alpha#1/2, sh-AP-2alphaA#1/2 and the negative control (sh-NC), were constructed at Genechem (Shanghai, China). MicroRNA (miRNA) mimics, including miR-4262 mimics and miR-185-5p mimics, with NC mimics as the control, were obtained from Genechem. For mRNA or circRNA overexpression, the sequence of YY1 or CTNNB1 was cloned into pcDNA3.1 vector (Genechem), while circAGFG1 sequence was cloned into pcDNA3.1(+) circRNA Mini vector (Genechem), constructing overexpression plasmids pcDNA3.1/YY1, pcDNA3.1/CTNNB1, and pcDNA3.1/circAGFG1, with empty vectors as the controls. Lipofectamine 2000 (Invitrogen) was adopted for cell transfection for 48 h.

### Cell counting Kit-8 (CCK-8) assay

After 0, 24, 48, 72 or 96 h of incubation, cells in 96-well plates were treated with CCK-8 reagent (Dojindo, Kumamoto, Japan) for 2 h at 37 °C. Cell viability was determined via evaluating the optical density (OD) value at 450 nm with a microplate reader (Bio-Rad, Hercules, CA, USA).

### Colony formation assay

Clonogenic cells in 6-well plates were cultured for 14 days and then stained with 0.5% crystal violet in methanol for 15 min. Colonies containing more than 50 cells were counted.

### 5-Ethynyl-2′-deoxyuridine (EdU) incorporation assay

Cells in 24-well plates were laid on sterile coverslips. EdU kit (RiboBio, Guangzhou, China) was used for labeling cells following the user’s guidebook. Nuclei were stained with 4′,6-diamidino-2-phenylindole (DAPI; Beyotime, Shanghai, China). Images were captured by laser confocal microscopy (Olympus, Tokyo, Japan).

### Flow cytometry

Cells were reaped and washed in phosphate-buffered saline (PBS), followed by double-staining with Annexin V-propidium iodide (PI) (Invitrogen). A flow cytometer (Beckman Coulter, Brea, CA, USA) was utilized for detecting apoptotic cells using FCS Express 3.0 software (De Novo Software, Glendale, CA, USA). For the detection of CD133^+^ cells, phycoerythrin (PE)-conjugated anti-CD133 antibody (Invitrogen) was incubated with cells for 1 h at 4 °C prior to flow cytometry analysis.

### Terminal deoxynucleotidyl transferase-mediated dUTP nick end labeling (TUNEL) assay

The fixed cells were permeabilized and treated with TUNEL detecting solution (Clontech, Mountain View, CA, USA) and Hoechst 33342 solution (Sigma-Aldrich). Cells were finally observed under a fluorescence microscope (Nikon, Tokyo, Japan).

### Transwell assay

The 24-well-plate transwell chamber (Corning Incorporated, Corning, NY, USA) pre-coated with Matrigel was for invasion assay, while the transwell chamber without Matrigel was for migration assay. Cells were cultured in the upper chamber and were allowed to transfer to the lower chamber with complete medium. Invaded and migrated cells were fixed in methanol, dyed with crystal violet, and counted under a microscope (Leica, Wetzlar, Germany).

### Sphere formation assay

Cells were cultivated in 96-well Clear Round Bottom Ultra Low Attachment Microplate (Corning). After 7 days, the tumor spheres formed in the microplate were observed and counted. The percentage of cells which could develop into a tumor sphere was regarded as sphere formation efficiency.

### Western blotting

Total cellular proteins were extracted using radioimmunoprecipitation assay (RIPA) buffer (Thermo Scientific), separated using sodium dodecyl sulfate-polyacrylamide gel electrophoresis (SDS-PAGE), and transferred to polyvinylidene fluoride (PVDF) membrane (Millipore, Billerica, MA, USA). The membrane was blocked using fat-free milk and incubated with specific antibodies at 4 °C overnight. The primary antibodies were all purchased from Abcam (Cambridge, MA, USA), including anti-Bax (ab32503), anti-Bcl-2 (ab185002), anti-MMP2 (ab215986), anti-CD44 (ab216647), anti-SOX2 (ab97959), anti-Oct4 (ab93689), anti-Nanog (ab218524), anti-β-catenin (ab32572), anti-cyclin D1 (ab1663), anti-c-myc (ab32072), and anti-GAPDH (ab8245). Membranes were then cultivated with secondary antibody (ab205718; Abcam) and detected by chemiluminescence system (Bio-Rad).

### Tumor xenograft assay

Transfected cells were subcutaneously inoculated into BALB/c male nude mice (National Laboratory Animal Center, Beijing, China). Mice were randomly divided into two groups. Tumor volume was calculated every 4 days. Four weeks later, mice were sacrificed before tumors were excised and weighed. This assay was performed strictly in line with the protocol approved by the Animal Research Ethics Committee of Affiliated Hangzhou First People’s Hospital, Zhejiang University School of Medicine.

### Immunohistochemistry (IHC)

Paraffin-embedded tissues from xenograft assay were sectioned at 4-μm thick and cultured with primary antibodies including anti-Ki67, anti-CD44, anti-SOX2, anti-Oct4, and anti-Nanog (Santa Cruz Biotechnology, Dallas, TX, USA) at 4 °C overnight, and secondary antibodies were applied for 30 min of treatment at room temperature.

### Hematoxylin and eosin (HE) staining

Paraffin-embedded sections were fixed, dehydrated and dried at 45 °C. Sections were then subjected to HE staining (Sigma-Aldrich), followed by observation.

### Luciferase reporter assay

For detecting Wnt/β-catenin signaling (TOP/FOP assay), TOP/FOP-flash luciferase reporter vectors (Millipore) were co-transfected into cells with sh-circAGFG1#1 or sh-NC. For CTNNB1 promoter analysis, pGL3 luciferase vectors (Promega, Madison, WI, USA) harboring the sequences of wild-type or mutant CTNNB1 promoter (pGL3-CTNNB1-Pro-Wt/Mut-Luc) were co-transfected into cells with sh-circAGFG1#1 or sh-NC, or with pcDNA3.1/YY1 or pcDNA3.1 empty vector. For miRNA interaction analysis, the sequence of cirAGFG1 or YY1 3′-UTR with wild-type or mutant binding sites of miR-4262 or miR-185-5p were sub-cloned into pmirGLO vectors (Promega). The constructed cirAGFG1-Wt/Mut or YY1-Wt/Mut luciferase reporters were co-transfected with the indicated plasmids into cells. Luciferase activity was determined by Luciferase Reporter System (Promega).

### Immunofluorescence (IF)

Cells in 6-well plates were fixed, rinsed in PBS and cultivated with antibody against β-catenin (Abcam) for 1.5 h at room temperature. After treatment with secondary antibody, nuclei were stained with Hoechst 33342 solution (Sigma-Aldrich) for 10 min, and cells were visualized under a fluorescence microscope (Olympus).

### Subcellular fractionation

Nuclear and Cytoplasmic Extraction Reagents (Thermo Scientific) were applied to isolate the nuclei and cytoplasm of cells. qRT-PCR was performed to quantify circAGFG1, U6 (the nuclear control) and GAPDH (the cytoplasmic control) in different fractions.

### DNA pull-down

The sequence of CTNNB1 promoter was synthesized in vitro and labeled with biotin by GenePharma (Shanghai, China). The biotin-labeled DNA was co-incubated with nuclear extract and streptavidin agarose magnetic beads (Invitrogen). After washing, the remaining proteins binding to CTNNB1 promoter were eluted and subjected to mass spectrometry or western blot. The antibodies (Abcam) against various transcription factors were applied.

### RNA pull-down

The probes for circAGFG1 (GenePharma) with or without biotin labeling were synthesized in vitro. Then the probes were co-incubated with cell lysates and streptavidin agarose magnetic beads (Invitrogen). After elution, the enrichment of specific miRNAs was assessed by qRT-PCR.

### Chromatin immunoprecipitation (ChIP)

Magna ChIP Chromatin Immunoprecipitation Kit (Millipore) was applied for ChIP assay. Chromatin was crosslinked with formaldehyde, sonicated into 200–500-bp fragments and immunoprecipitated with anti-YY1 or anti-IgG antibodies (Abcam) conjugated on magnetic beads. After washing and de-crosslinking, the enrichment of specific fragment was assayed by qRT-PCR.

### RNA immunoprecipitation (RIP)

Magna RIP RNA-Binding Protein Immunoprecipitation Kit (Millipore) was applied for RIP assay. Cell lysates obtained using RIP lysis buffer were mixed with magnetic beads coated by anti-Ago2 antibody (Abcam) or anti-IgG (Abcam) as the control in RIP buffer at 4 °C for 4 h. The enrichment of specific RNAs was measured using qRT-PCR.

### Statistical analysis

Data with normal distribution were obtained from three independent experiments and shown as mean ± standard deviation (SD). Prism 6.0 software (GraphPad, La Jolla, CA, USA) and SPSS 18.0 software (SPSS, Chicago, IL, USA) were used for statistical analyses, with *p* < 0.05 as the threshold of significance. Group differences were analyzed using Student’s *t*-test or analysis of variance (ANOVA). Pearson’s correlation analysis was applied for gene expression correlation.

## Results

### CircAGFG1 was upregulated in CRC cell lines

**Fig. 1:**
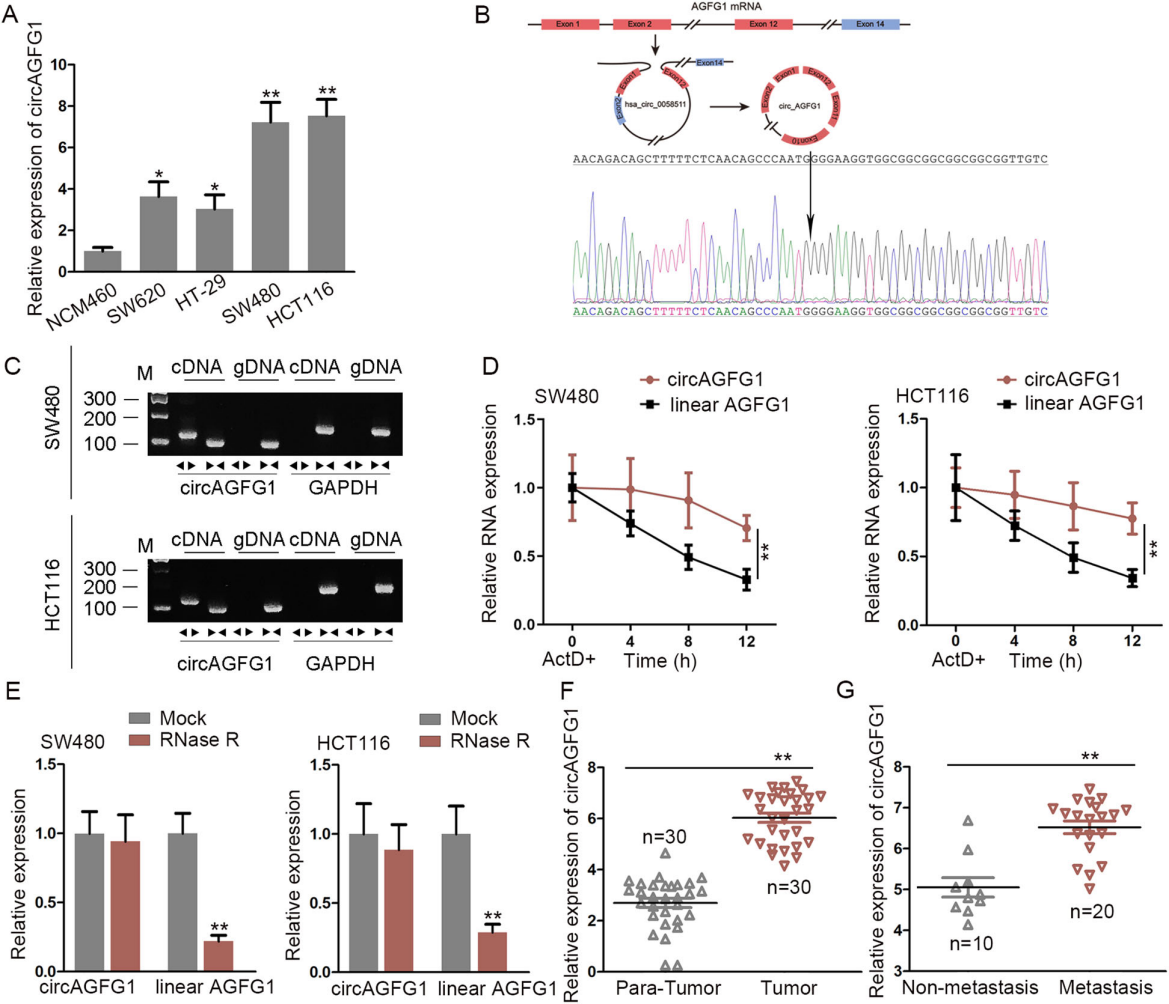
CircAGFG1 expression was upregulated in CRC cell lines. **a** The expression of circAGFG1 in CRC cell lines and in normal cells was assessed by qRT-PCR. Two CRC cell lines with higher levels of circAGFG1 were adopted for subsequent experiments. **b** A schematic diagram of the genomic location and splicing pattern of circAGFG1. The back-splicing site was verified by Sanger sequencing. **c** The existence of circAGFG1 was verified by PCR analysis and gel electrophoresis using convergent or divergent primers. **d** After ActD treatment, the expression of remaining circAGFG1 or AGFG1 mRNA in CRC cells at different time points was detected by qRT-PCR. **e** The stability of circAGFG1 under RNase R digestion was higher than that of linear AGFG1. **f** The expression of circAGFG1 was measured in 30 pairs of CRC tumor tissues and para-tumor tissues. **g** The expression of circAGFG1 in tumor tissues from CRC patients without liver metastasis (*n* = 10) or with liver metastasis (*n* = 20). We repeated the experiments three times to ensure the accuracy of the experiments. ^*^P < 0.05, ^**^P < 0.01.

First, we tested the expression of circAGFG1 in CRC cell lines (SW480, HCT116, SW620, and HT-29) and human colon mucosal epithelial cell line (NCM460). Results indicated that circAGFG1 was upregulated in CRC cell lines compared with NCM460 cell line (Fig. 1a). The structure diagram and the back-splicing site of circAGFG1 were displayed in Fig. 1b. To confirm the existence of circAGFG1, PCR analysis followed by gel electrophoresis was performed. Results indicated that the fragment between the divergent primers of circAGFG1 could be amplified in cDNA group, not in gDNA group (Fig. 1c). Besides, in CRC cells treated with ActD, the stability of circAGFG1 was higher than that of AGFG1 mRNA (Fig. 1d). Furthermore, we observed that circAGFG1, rather than AGFG1 mRNA, resisted the digestion of RNase R (Fig. 1e). It was also found that the expression of circAGFG1 was conspicuously elevated in CRC tumor tissues in comparison to para-tumor tissues (Fig. 1f). Besides, circAGFG1 expression in tumor tissues from CRC patients with liver metastasis was higher than that in those without liver metastasis (Fig. 1g). These results above confirmed the circular characteristics and high level of circAGFG1 in CRC.

### CircAGFG1 silencing significantly suppressed cancer progression in CRC cells

**Fig. 2:**
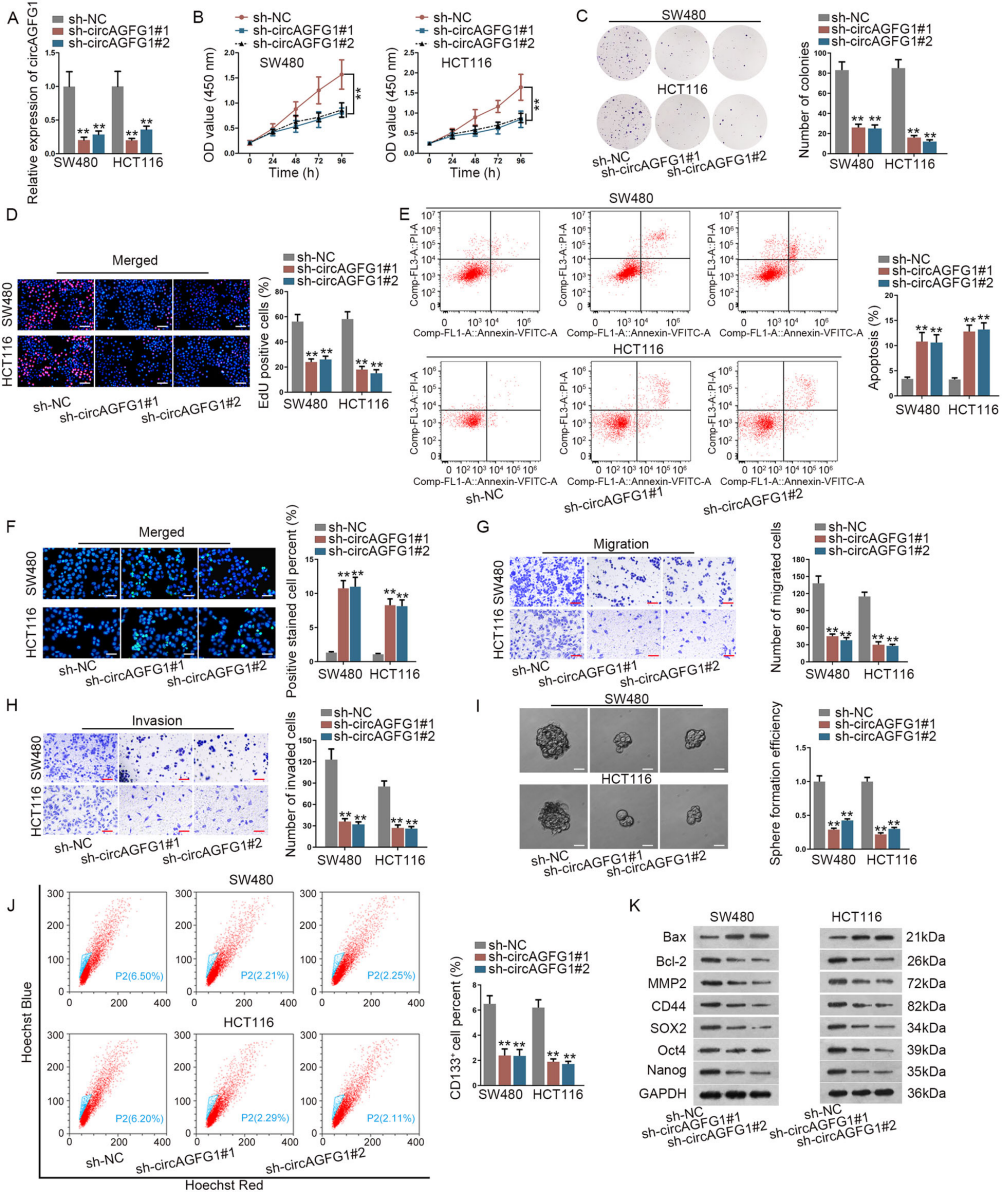
CircAGFG1 silencing significantly suppressed CRC cell proliferation, migration, invasion and stemness, and promoted cell apoptosis. **a** The expression of circAGFG1 was measured after the transfection of sh-NC, sh-circAGFG1#1 or sh-circAGFG1#2 into SW480 and HCT116 cells. **b**–**d** Cell proliferation was detected by CCK-8 assay (**b**), colony formation assay (**c**), and EdU assay (**d**) in CRC cells transfected with sh-NC or sh-circAGFG1#1/#2 (scale bar = 100 μm). **e**, **f** Cell apoptosis was examined by flow cytometry (**e**) and TUNEL assay (**f**) in each group (scale bar = 50 μm). **g**, **h** Cell migration (**g**) and invasion (**h**) were assessed by transwell assay in each group (scale bar = 70 μm). **i** Sphere formation efficiency was reduced after circAGFG1 silencing as shown in sphere formation assay (scale bar = 30 μm). **j** The percentage of CD133^+^ cells were reduced upon circAGFG1 knockdown as shown in flow cytometry assay. **k** The protein levels of Bax, Bcl-2, MMP2, CD44, SOX2, Oct4, and Nanog were detected by western blot in each group. We repeated the experiments three times to ensure the accuracy of the experiments. ^**^P < 0.01.

To explore the underlying effect of circAGFG1 on CRC progression, loss-of-function assays were conducted. The knockdown efficiency of circAGFG1 in SW480 and HCT116 cells was detected by qRT-PCR (Fig. 2a). CCK-8, colony formation and EdU assays revealed that cell proliferation was observably reduced after circAGFG1 silencing (Fig. 2b–d). Meanwhile, cell apoptosis was elevated by circAGFG1 knockdown (Fig. 2e, f). Besides, cell migration and invasion were hampered by circAGFG1 knockdown (Fig. 2g, h). In addition, sphere formation efficiency was decreased after circAGFG1 knockdown (Fig. 2i). CD133 (a typical marker for stemness) positive cell proportion also declined after circAGFG1 silencing (Fig. 2j). Cell apoptosis related proteins (Bax and Bcl-2), migration-related protein (MMP2) and stemness-related proteins (CD44, SOX2, Oct4, and Nanog) were detected by western blot. The results indicated that Bax expression was increased, and Bcl-2, MMP2, CD44, SOX2, Oct4, and Nanog expressions were decreased in sh-circAGFG1#1/2 groups compared to sh-NC group (Fig. 2k). Collectively, circAGFG1 silencing significantly suppressed cell proliferation, migration, invasion and stemness, and promoted cell apoptosis in CRC.

### CircAGFG1 accelerated CRC tumorigenesis and metastasis in vivo

**Fig. 3:**
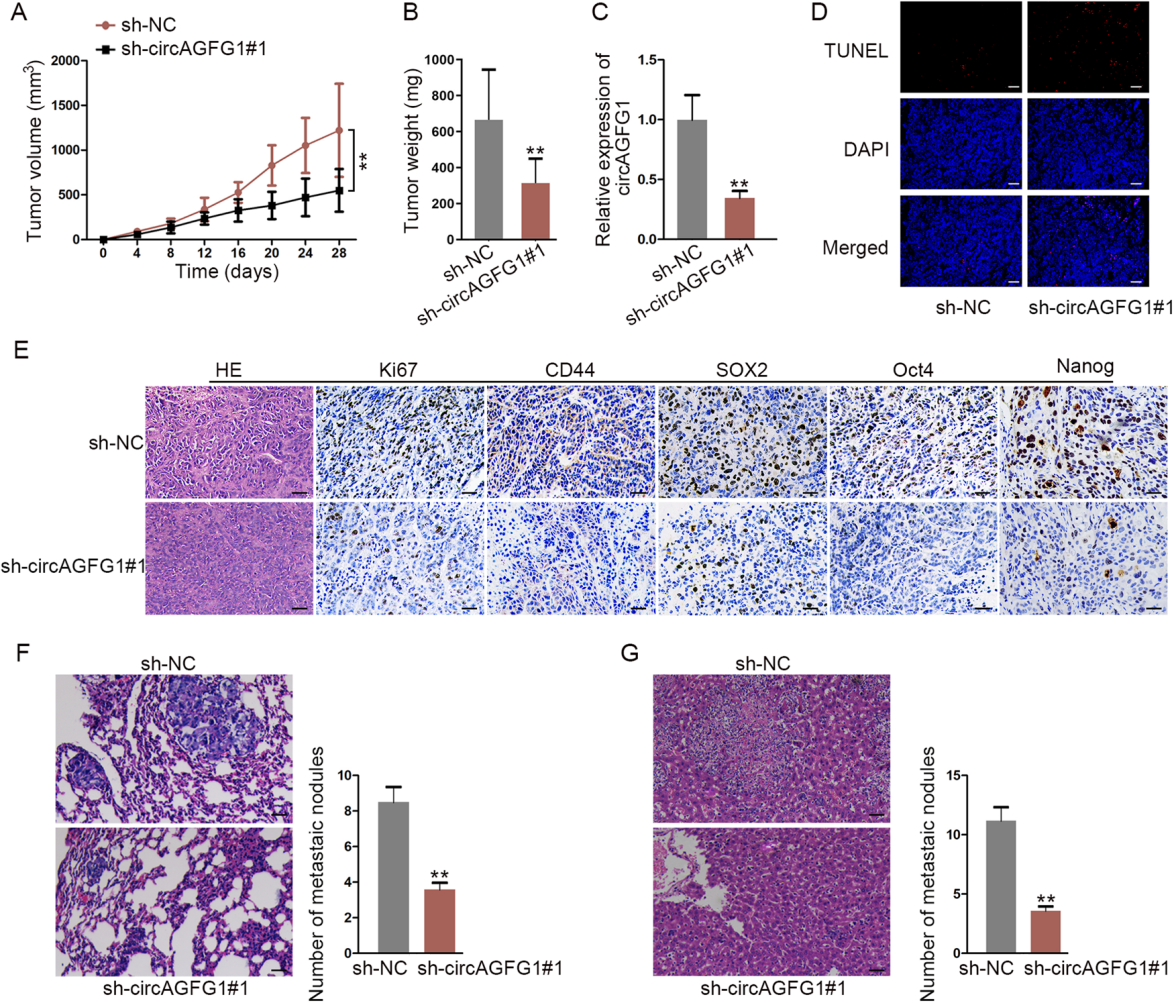
CircAGFG1 accelerated CRC tumor growth and metastasis in vivo. **a** The growth curve illustrated the change of tumor volume in sh-NC group or sh-circAGFG1#1 group. **b** The weight of tumor from sh-NC group or sh-circAGFG1#1 group was measured. **c** The expression of circAGFG1 in excised tumors of each group was assessed by qRT-PCR. **d** Apoptosis was detected by TUNEL assay in each group (scale bar = 100 μm). **e** Tumor tissues of each group were subjected to HE staining. The expressions of Ki67, CD44, SOX2, Oct4, and Nanog were examined by IHC assay (scale bar = 100 μm). **f**, **g** After HE staining, the lung metastatic nodules (**f**, scale bar = 100 μm) or liver metastatic nodules (**g**, scale bar = 100 μm) of each group were counted. We repeated the experiments three times to ensure the accuracy of the experiments. ^**^P < 0.01.

To verify the role of circAGFG1 in CRC tumor growth and liver or lung metastasis, in vivo assays were carried out. SW480 cells transfected with sh-NC or sh-circAGFG1#1 were subcutaneously injected into nude mice. As we observed, the volume and weight of tumors were both markedly reduced by circAGFG1 silencing (Fig. 3a, b). In addition, the expression of circAGFG1 in excised tumors was lower in sh-circAGFG1#1 group compared to sh-NC group (Fig. 3c). Results from TUNEL assay showed that apoptosis was increased in sh-circAGFG1#1 group (Fig. 3d). IHC assay demonstrated that the positivity of Ki67 (a cell proliferation indicator), CD44, SOX2, Oct4 and Nanog was all decreased after circAGFG1 knockdown (Fig. 3e). We also found that the number of lung metastatic nodules was reduced in sh-circAGFG1#1 group compared to sh-NC group (Fig. 3f). Furthermore, liver metastatic nodule number was also reduced by circAGFG1 silencing (Fig. 3g). Taken together, circAGFG1 could accelerate CRC tumorigenesis and metastasis in vivo.

### CircAGFG1 promoted CTNNB1 transcription in CRC cells

**Fig. 4:**
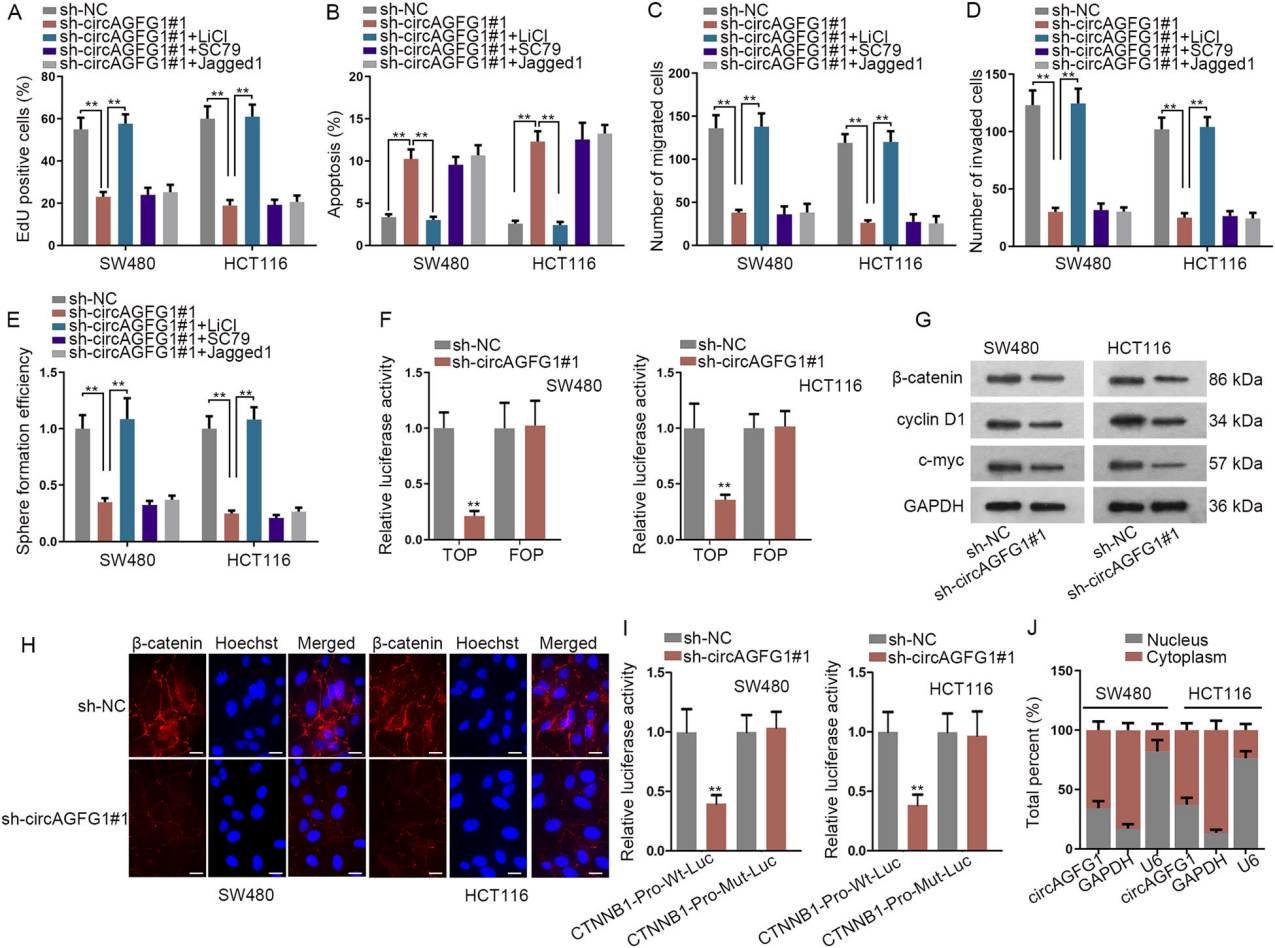
CircAGFG1 promoted CTNNB1 transcription in CRC cells. **a**–**e** The proliferation, apoptosis, migration, invasion and stemness of SW480 and HCT116 cells were respectively detected through EdU assay (**a**), flow cytometry (**b**), transwell assay (**c**, **d**) and sphere formation assay (**e**) in sh-NC, sh-circAGFG1#1, sh-circAGFG1#1+LiCl, sh-circAGFG1#1+SC79 or sh-circAGFG1#1+Jagged1 group. **f** TOP/FOP-flash assay was performed to verify the activity of Wnt/β-catenin pathway in cells transfected with sh-NC or sh-circAGFG1#1. FOP-flash reporter was adopted as the control of TOP flash reporter. **g** The protein expressions of β-catenin, cyclin D1 and c-myc were measured by western blot in sh-NC or sh-circAGFG1#1 group. **h** The nuclear translocation of β-catenin in sh-NC or sh-circAGFG1#1 group was assessed by IF assay (scale bar = 20 μm). **i** Luciferase reporter assay was performed to verify the effect of circAFGF1 knockdown on the activity of CTNNB1 promoter. **j** The cellular distribution of circAGFG1 was verified by subcellular fraction assay. We repeated the experiments three times to ensure the accuracy of the experiments. ^**^P < 0.01.

Evidence showed the participation of various signaling pathways in CRC progression, such as Wnt/β-catenin pathway^24^, AKT pathway^25^, and Notch pathway^26^. To explore the downstream signaling by which circAGFG1 could exert its function, Wnt/β-catenin pathway activator (LiCl), AKT pathway activator (SC79) and Notch pathway activator (Jagged1) were used for rescue assays. We found that only LiCl could notably rescue the inhibited proliferation, migration, invasion and stemness, and the facilitated apoptosis of CRC cells caused by circAGFG1 knockdown (Fig. 4a–e). These results showed that circAGFG1 could regulate Wnt/β-catenin pathway. Results of TOP/FOP-flash assay showed that the luciferase activity of TOP flash reporters was reduced by knocking down circAGFG1 (Fig. 4f). Western blot assay displayed that the protein levels of Wnt/β-catenin pathway-related factors (β-catenin, cyclin D1 and c-myc) were decreased by circAGFG1 knockdown (Fig. 4g). Through IF assay, we found that the nuclear translocation of β-catenin was inhibited after circAGFG1 knockdown (Fig. 4h). Furthermore, the luciferase activity of CTNNB1-Pro-Wt-Luc reporter was reduced by knocking down circAGFG1, while the luciferase activity of CTNNB1-Pro-Mut-Luc reporter was not affected (Fig. 4i), indicating that CTNNB1 transcription could be regulated by circAGFG1. However, circAGFG1 was located in the cytoplasm of CRC cells (Fig. 4j). All data suggested that circAGFG1 activated Wnt/β-catenin signaling by facilitating CTNNB1 transcription in CRC cells, whereas owing to its cytoplasmic distribution, circAGFG1 could not directly regulate CTNNB1 at transcriptional level.

### YY1 transcriptionally facilitated CTNNB1 in CRC cells

**Fig. 5:**
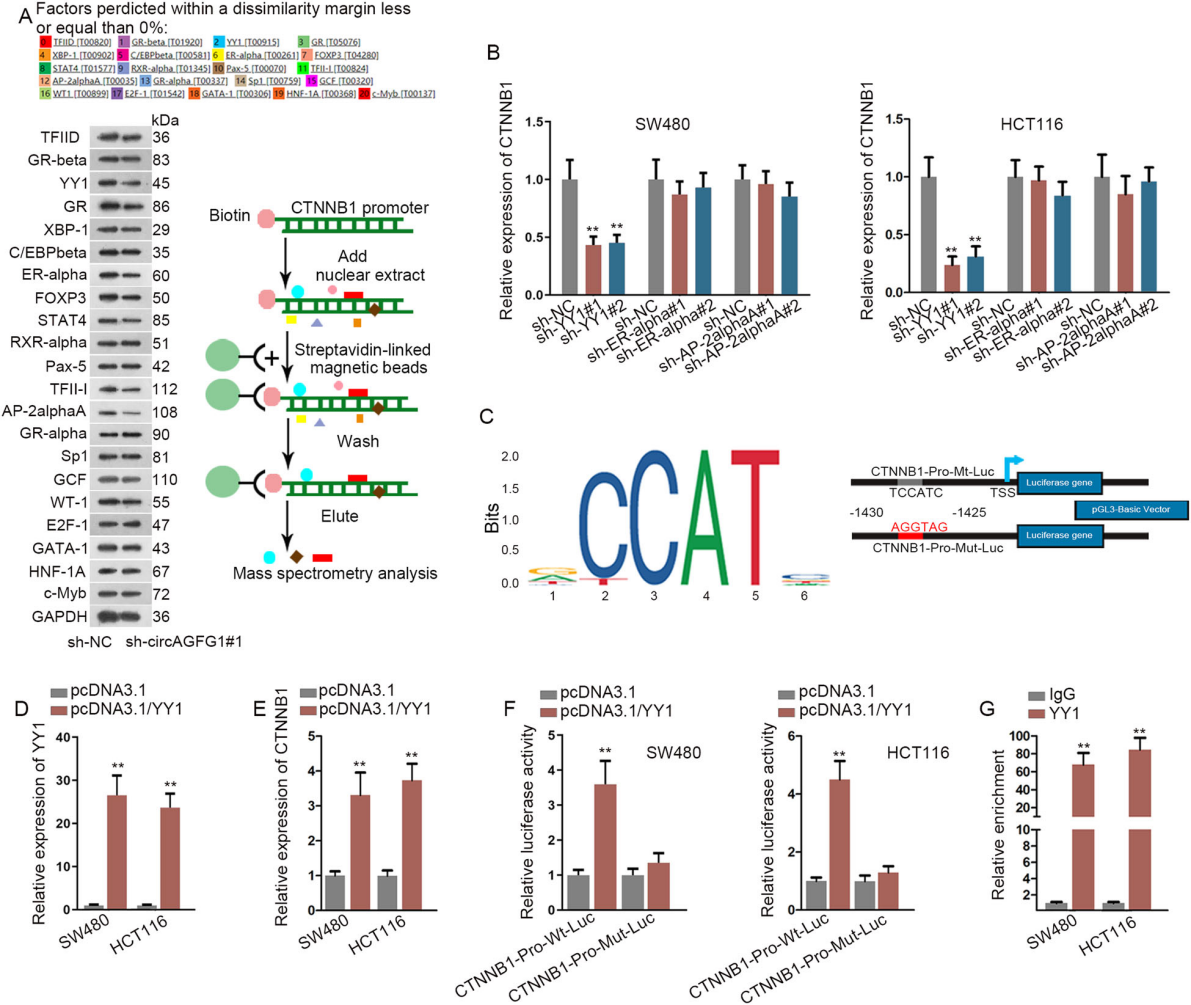
YY1 transcriptionally facilitated CTNNB1 expression in CRC cells. **a** PROMO database (http://alggen.lsi.upc.es/cgi-bin/promo_v3/promo/promoinit.cgi?dirDB=TF_8.3/) was adopted to predict the potential transcription factors for CTNNB1. The enrichment of these transcription factors on CTNNB1 promoter in response to circAGFG1 knockdown was illustrated using DNA pull-down assay followed by western blot. The schematic diagram of DNA pull-down assay was depicted. **b** The expression of CTNNB1 was detected by qRT-PCR in CRC cells transfected with shRNAs targeting YY1, ER-alpha and AP-2alphaA. **c** The binding motif of YY1 was depicted according to JASPAR database (http://jaspar.genereg.net/). The sequence of CTNNB1 promoter with a putative YY1 binding site (TCCATC) or a mutated binding site (AGGTAG) was cloned into pGL3 vector for luciferase reporter assay. **d**, **e** After overexpressing YY1, the promoted expression of YY1 (**d**) and CTNNB1 (**e**) was detected by qRT-PCR. **f** Luciferase reporter assay was performed to verify the interaction between YY1 and CTNNB1 promoter. **g** ChIP assay was conducted to confirm the binding ability between YY1 and CTNNB1 promoter. We repeated the experiments three times to ensure the accuracy of the experiments. ^**^P < 0.01.

To determine how CTNNB1 transcription was regulated by circAGFG1, we conducted DNA pull-down assay and found that 21 transcription factors were enriched by the biotin-labeled probe of CTNNB1 promoter. Among them, the enrichment of YY1, ER-alpha or AP-2alphaA was decreased by circAGFG1 knockdown (Fig. 5a). Thus, YY1, ER-alpha, and AP-2alphaA were predicted as potential transcription factors for CTNNB1. With the application of shRNAs, the expressions of these transcription factors were separately knocked down (Fig. S1A). It was found that CTNNB1 expression was only decreased in sh-YY1#1/2 groups, suggesting that YY1 was responsible for CTNNB1 transcription (Fig. 5b). The binding motif of YY1 on CTNNB1 promoter was displayed in Fig. 5c. For further study, after the overexpression efficiency of YY1 in CRC cells was tested (Fig. 5d), it was observed that the expression of CTNNB1 was dramatically increased by YY1 overexpression (Fig. 5e). Moreover, the luciferase activity of CTNNB1-Pro-Wt-Luc reporter was promoted by overexpressing YY1, while no obvious change was shown in the luciferase activity of CTNNB1-Pro-Mut-Luc reporter (Fig. 5f). ChIP assay revealed that CTNNB1 promoter was significantly enriched in anti-YY1 group compared with anti-IgG group (Fig. 5g). These results displayed that circAGFG1 regulated CTNNB1 at transcriptional level through YY1.

### CircAGFG1 regulated YY1 expression through absorbing miR-4262 and miR-185-5p

**Fig. 6:**
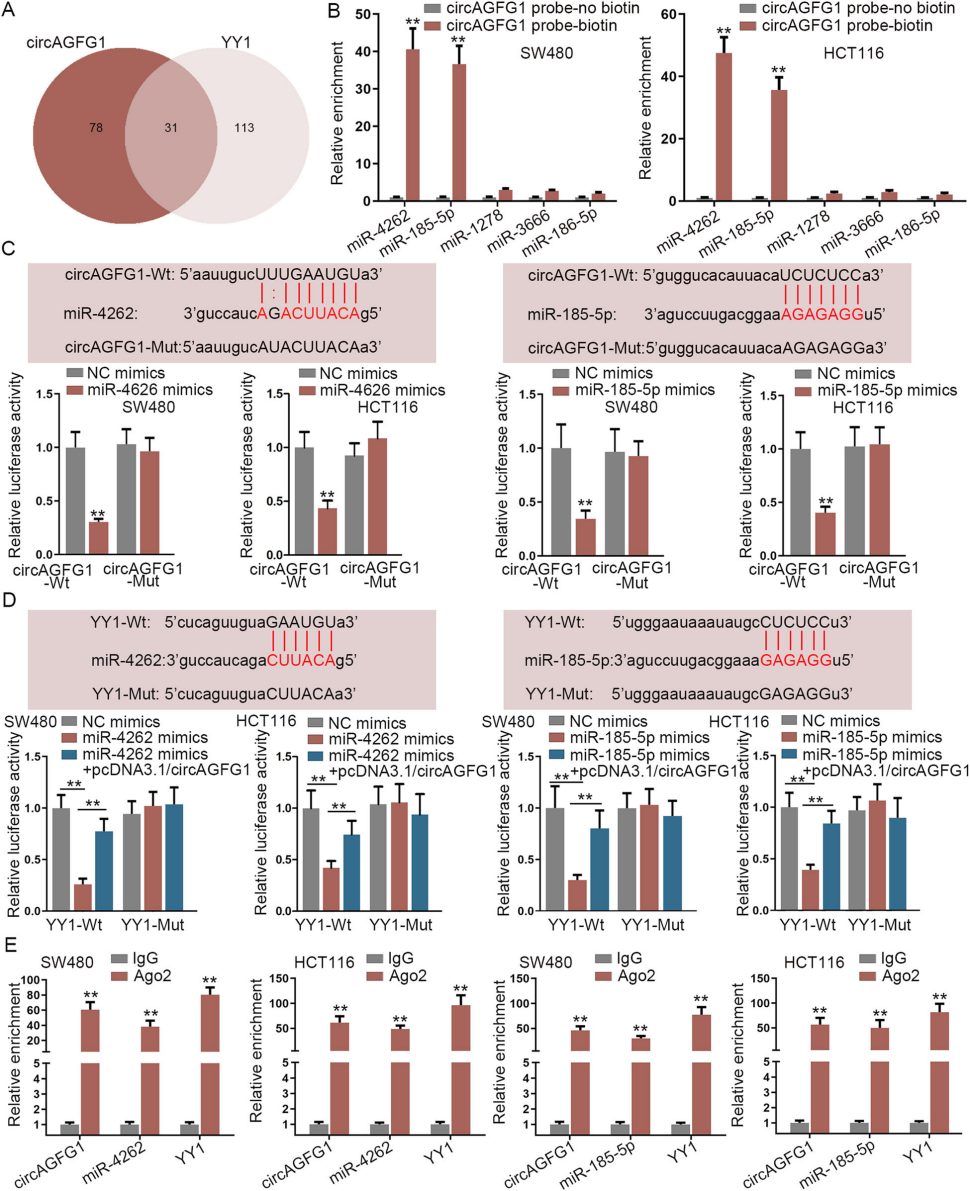
CircAGFG1 regulated YY1 expression through absorbing miR-4262 and miR-185-5p. **a** Venn diagram showed 31 miRNAs which could bind to both circAGFG1 and YY1 3′-UTR. **b** RNA pull-down assay was carried out to detect the binding capacity between circAGFG1 and miRNAs. **c** Luciferase reporter assay was performed to verify the interaction between circAGFG1 and miR-4262 or miR-185-5p. **d** Luciferase reporter assay was performed to verify the interaction between YY1 3′-UTR and miR-4262 or miR-185-5p, as well as the competitive effect of circAGFG1. **e** RIP assay was carried out to further confirm the enrichments of YY1, miR-4262, miR-185-5p and circAGFG1 in IgG group and Ago2 group. We repeated the experiments three times to ensure the accuracy of the experiments. ^**^P < 0.01.

Subsequently, we explored how circAGFG1 regulated YY1 in CRC. With the employment of bioinformatics tools, 31 miRNAs which could bind to circAGFG1 and YY1 3′-untranslated region (3′-UTR) were found (Fig. 6a). Then, specific probes for circAGFG1 were used for RNA pull-down assay. We found that miR-4262 and miR-185-5p were notably enriched in biotin-labeled circAGFG1 probe group (Fig. 6b). To investigate the potential interaction of miR-4262 and miR-185-5p with circAGFG1 or YY1, the transfection efficiency of miR-4262 mimics or miR-185-5p mimics into CRC cells was verified (Fig. S1B). The binding site between circAGFG1 or YY1 3′-UTR, and miR-4262 or miR-185-5p was presented. Through luciferase reporter assay, the luciferase activity of circAGFG1-Wt reporter was weakened by the upregulation of miR-4262 or miR-185-5p, while that of circAGFG1-Mut reporter had no obvious change (Fig. 6c). Besides, the luciferase activity of YY1-Wt reporter was reduced by miR-4262 mimics or miR-185-5p mimics, and this suppression was recovered by overexpressing circAGFG1 (Fig. 6d). Furthermore, RIP assay demonstrated that circAGFG1 and YY1 existed in miR-4262 or miR-185-5p-mediated RNA-induced silencing complex (Fig. 6e). These results suggested that circAGFG1 regulated YY1 expression through absorbing miR-4262 and miR-185-5p.

### CircAGFG1 was involved in CRC progression through modulating YY1/CTNNB1

**Fig. 7:**
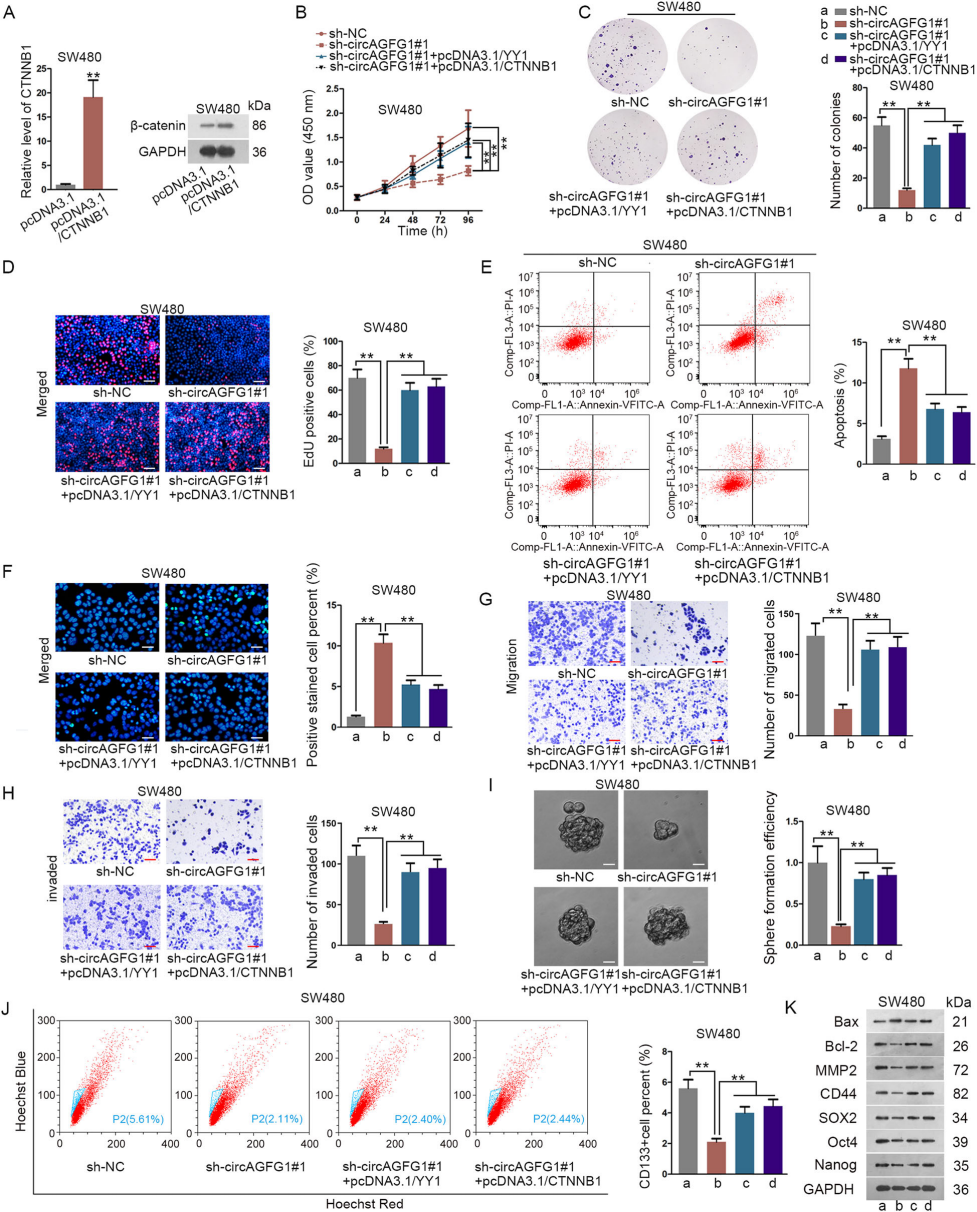
CircAGFG1 was involved in CRC progression through modulating YY1/CTNNB1. **a** The overexpression efficiency of CTNNB1 in SW480 cells was examined by qRT-PCR and western blot. **b**–**d** Cell proliferation was detected by CCK-8 assay (**b**), colony formation assay (**c**) and EdU assay (**d**) in each group (scale bar = 100 μm). **e**, **f** Cell apoptosis was examined by flow cytometry (**e**) and TUNEL assay (**f**) in each group (scale bar = 50 μm). **g**, **h** Cell migration (**g**) and invasion (**h**) were assessed by transwell assay in each group (scale bar = 70 μm). **i** Sphere formation efficiency was detected in each group by sphere formation assay (scale bar = 30 μm). **j** CD133^+^ cell percentage was assessed in each group by flow cytometry assay. **k** The protein levels of Bax, Bcl-2, MMP2, CD44, SOX2, Oct4, and Nanog in each group were detected by western blot. In this figure, “a, b, c and d” represents the group of sh-NC, sh-circAGFG1#1, sh-circAGFG1#1+pcDNA3.1/YY1 and sh-circAGFG1#1+pcDNA3.1/CTNNB1, respectively. We repeated the experiments three times to ensure the accuracy of the experiments. ^**^P < 0.01.

To further confirm whether circAGFG1 participated in regulating CRC progression through modulating YY1/CTNNB1, rescue assays were carried out. qRT-PCR and western blot validated the overexpressing efficiency of CTNNB1 in SW480 cells (Fig. 7a). As demonstrated, the inhibitory role of circAGFG1 knockdown on CRC cell proliferation was rescued by YY1 overexpression or CTNNB1 overexpression (Fig. 7b–d). Besides, cell apoptosis promoted by knocking down circAGFG1 was reversed by the ectopic expression of YY1 or CTNNB1 (Fig. 7e, f). In transwell assay, circAGFG1 silencing inhibited cell migration and invasion, while this effect was reversed by the upregulation of YY1 or CTNNB1 (Fig. 7g, h). Moreover, sphere formation efficiency was decreased after circAGFG1 silencing, and this effect was abolished after overexpression of YY1 or CTNNB1 (Fig. 7i). CD133^+^ cell proportion declined by the silencing circAGFG1 was restored after increased level of YY1 or CTNNB1 (Fig. 7j). Western blot assay showed that the effects of circAGFG1 knockdown on the levels of proteins associated with apoptosis, migration and stemness were rescued by overexpressing YY1 or CTNNB1 (Fig. 7k). In conclusion, circAGFG1 could aggravate CRC progression by regulating YY1/CTNNB1.

### CircAGFG1 activated WNT/β-catenin pathway through the axis of miR-4262 or miR-185-5p/YY1/CTNNB1

**Fig. 8:**
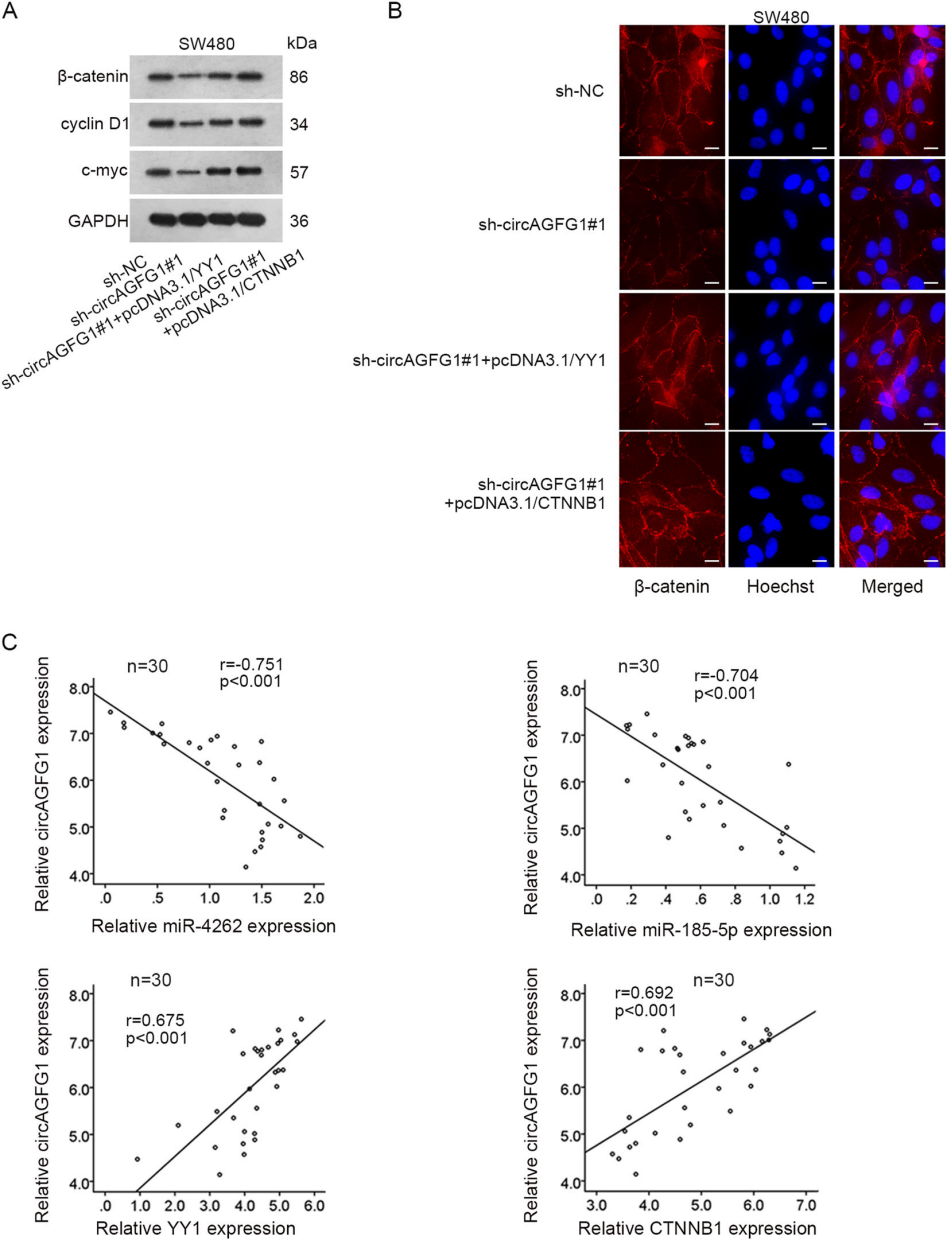
CircAGFG1 activated WNT/β-catenin pathway through the miR-4262/miR-185-5p/YY1/CTNNB1 axis. **a** The protein expressions of β-catenin, cyclin D1 and c-myc in the group of sh-NC, sh-circAGFG1#1, sh-circAGFG1#1+pcDNA3.1/YY1 or sh-circAGFG1#1+pcDNA3.1/CTNNB1 were measured by western blot. **b** The nuclear translocation of β-catenin in each group was assessed by IF assay (scale bar = 20 μm). **c** The expression association between circAGFG1 and miR-4262, miR-185-5p, YY1 or CTNNB1 was revealed by Pearson’s correlation analysis. We repeated the experiments three times to ensure the accuracy of the experiments. ^**^P < 0.01.

Finally, we probed whether circAGFG1 regulated YY1/CTNNB1 to activate WNT/β-catenin pathway. Through western blot assay, we observed that the decreased protein levels of β-catenin, cyclin D1 and c-myc in sh-circAGFG1#1-transfected SW480 cells were recovered by the overexpression of YY1 or CTNNB1 (Fig. 8a). Through IF assay, the nuclear translocation of β-catenin was uncovered to be reduced in circAGFG1 knockdown group, while this effect was reversed by YY1 or CTNNB1 overexpression (Fig. 8b). Finally, we found that the expression of circAGFG1 was negatively associated with miR-4262 or miR-185-5p and positively correlated with YY1 or CTNNB1 (Fig. 8c). Taken together, circAGFG1 activated WNT/β-catenin pathway in CRC through regulating miR-4262 or miR-185-5p/YY1/CTNNB1 axis.

## Discussion

circRNAs have been confirmed to play a regulatory role in cancer progression, including CRC^9–12^. It has been reported that circAGFG1 is involved in the regulation of triple-negative breast cancer and non-small-cell lung cancer^15,16^; nevertheless, its role in CRC is still unclear. In our study, we discovered that circAGFG1 was highly expressed in CRC cell lines. Functional assays revealed that circAGFG1 silencing significantly suppressed CRC cell proliferation, migration, invasion and stemness, and promoted cell apoptosis. Meanwhile, we found that circAGFG1 also exacerbated CRC tumorigenesis and metastasis in vivo. These findings indicated that circAGFG1 functioned as an oncogene in CRC.

Plenty of evidence showed that the tumorigenesis of CRC is attributed to the activation of Wnt/β-catenin signaling pathway^19–21^. CTNNB1 is the gene encoding β-catenin, the pivotal factor in Wnt/β-catenin signaling pathway^22,23^. Here, we found that the effect of circAGFG1 knockdown on cancer progression in CRC cells could be rescued by the treatment of LiCl. In addition, circAGFG1 knockdown reduced the expression levels of Wnt/β-catenin pathway factors, and hampered the translocation of β-catenin into nucleus. Importantly, circAGFG1 silencing decreased the transcriptional activity of CTNNB1, indicating the regulatory role of circAGFG1 on Wnt/β-catenin signaling pathway. The cytoplasmic localization of circAGFG1 suggested that circAGFG1 could not directly regulate CTNNB1 at transcriptional level. We probed whether circAGFG1 could regulate the expression of transcription factors which could activate CTNNB1 transcription. Interestingly, YY1 was found to be down-regulated by circAGFG1 knockdown, and have the potential to interact with the promoter region of CTNNB1. It has been indicated that YY1 acts as an important transcription factor to affect critical gene expression. For example, YY1 promotes tumorigenesis via regulating glucose transporter GLUT3^27^. YY1 enhances G6PD transcription and directly activates the pentose phosphate pathway to accelerate cell proliferation^28^. YY1 facilitates lung cancer progression by promoting lncRNA-PVT1 transcription^29^. Similarly, we found that YY1 functioned as a transcription factor to induce the transcription of CTNNB1 in CRC cells.

MiRNAs are a type of non-coding RNAs with 22–25 nucleotides in length and have been reported as key regulators of gene expression in cancers^30,31^. For instance, miR-483-5p and miR-139-5p enhance adrenocortical cancer aggressiveness by regulating N-myc downstream-regulated genes^32^. Oncogenic miR-19a and miR-19b promote lung cancer cell proliferation and migration by regulating tumor suppressor MTUS1^33^. Exosomal miR-423-5p acts as a new marker in gastric cancer and drove cancer growth and metastasis by targeting SUFU^34^. Besides, miRNAs can be involved in the regulation of CRC development. Downregulation of miR-9 facilitated EMT in CRC cells through regulating ANO1^35^. Upregulated miR-1258 directly targets E2F8 to regulate cell cycle and inhibit cell proliferation in CRC^36^. MiR-182 facilitated cell proliferation and tumor growth in CRC by modulating DAB2IP^37^. In our study, miR-4262 and miR-185-5p was found to be able to interact with circAGFG1 and YY1. These two miRNAs have been reported as tumor-suppressors in multifarious cancers. MiR-4262 targeted CD163 to inhibit cell proliferation and invasion in gastric cancer, and low miR-4262 level predicts poor prognosis^38^. MiR-4262 contributed to cell apoptosis and inhibited proliferation of colon cancer cells via the involvement of GALNT4^39^. Nevertheless, the role of miR-4262 has not been reported in CRC. MiR-185-5p inhibits cell migration and invasion of hepatocellular carcinoma via targeting ROCK2^40^. LncRNA FOXD2-AS1 promotes CRC growth through sponging miR-185-5p^41^. Herein, mechanistic analysis revealed that circAGFG1 directly sponged miR-4262 and miR-185-5p to regulate YY1 expression. Eventually, rescue assays demonstrated that the effect of circAGFG1 silencing in CRC progression was observably restored by up-regulating YY1 or CTNNB1.

In brief, our study is the first to research circAGFG1/miR-4262 or miR-185-5p/YY1/CTNNB1 axis in CRC. Our results revealed that circAGFG1 up-regulated YY1 to activate CTNNB1 transcription and thereby accelerated metastasis and stemness in CRC by sponging miR-4262 and miR-185-5p. These findings provided a novel insight into the exploration on therapeutic targets in CRC.

## Supplementary information

Figure S1

supplementary figure legends
