# Supplementary figures and images for: CircAGFG1 drives metastasis and stemness in colorectal cancer by modulating YY1/CTNNB1
Source: Cell Death Dis. 2020 Jul 17;11(7):542. doi: 10.1038/s41419-020-2707-6 (PMC7367849; doi:10.1038/s41419-020-2707-6)

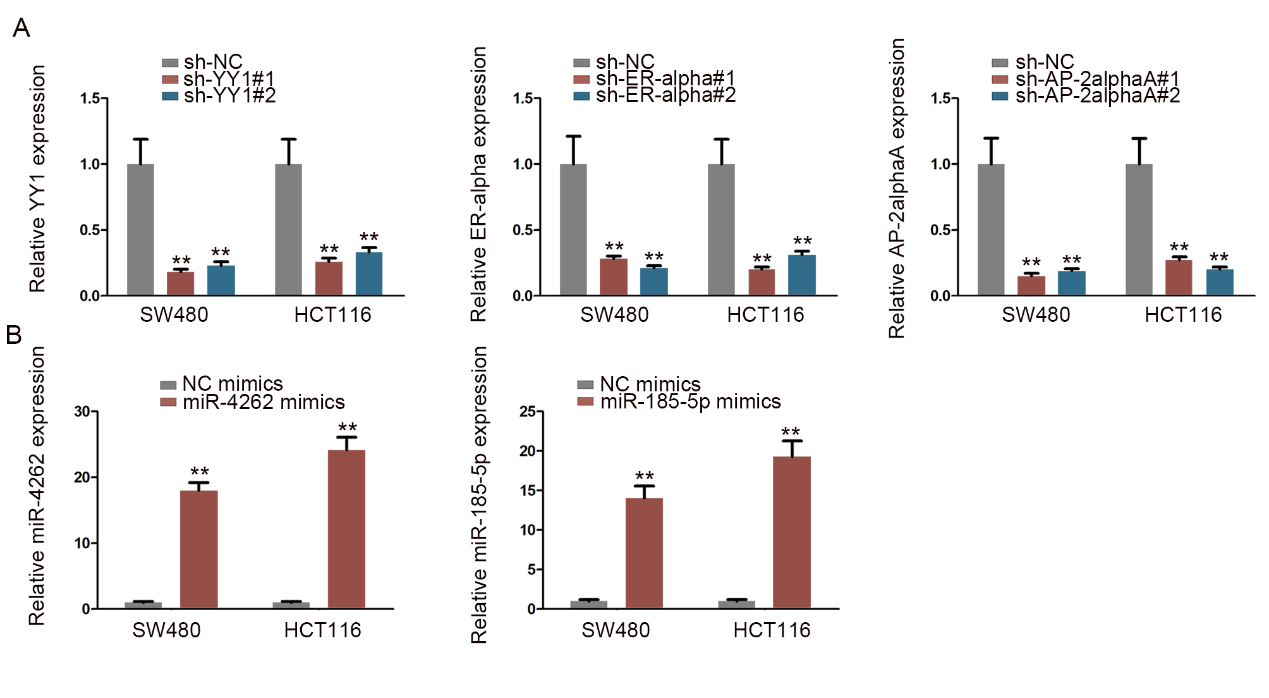

Supplement: Supplementary file 1 — Figure S1 [file 41419_2020_2707_MOESM1_ESM.tif]
