# Supplementary material for: CircAGFG1 drives metastasis and stemness in colorectal cancer by modulating YY1/CTNNB1
Source: Cell Death Dis. 2020 Jul 17;11(7):542. doi: 10.1038/s41419-020-2707-6 (PMC7367849; doi:10.1038/s41419-020-2707-6)
Supplement: Supplementary file 2 — supplementary figure legends [file 41419_2020_2707_MOESM2_ESM.docx]

**Supplementary figure 1**

(A) The knockdown efficiency of YY1, ER-alpha and AP-2alphaA was detected by qRT-PCR assay. (B) The overexpression efficiency of miR-4262 and miR-185-5p was examined by qRT-PCR assay. We repeated the experiments three times to ensure the accuracy of the experiments. ^**^*P* < 0.01.
